# Low-Latency Oriented Joint Data Compression and Resource Allocation in NOMA-MEC Networks: A Deep Reinforcement Learning Approach

**DOI:** 10.3390/s26010285

**Published:** 2026-01-02

**Authors:** Fangqing Tan, Yu Zeng, Chao Lan, Zou Zhou

**Affiliations:** Key Laboratory of Cognitive Radio and Information Processing of Ministry of Education, Guilin University of Electronic Technology, Guilin 541004, China

**Keywords:** mobile edge computing, non-orthogonal multiple access, data compression, resource allocation, deep reinforcement learning

## Abstract

To alleviate communication pressure and terminal resource constraints in mobile edge computing (MEC) networks, this paper proposes a resource allocation optimization method for MEC systems that integrates data compression technology and non-orthogonal multiple access technology. This method considers practical constraints such as terminal device battery capacity and computational resource limitations. By jointly optimizing computational resource allocation, task offloading strategies, and data compression ratios, it constructs an optimization model aimed at minimizing the total task processing latency. Addressing the challenges stemming from the non-convex nature of the problem and the dynamic variations in channel conditions and task requirements, this paper proposes a softmax deep double deterministic policy gradient algorithm, where softmax operator function mitigates both overestimation and underestimation biases inherent in traditional reinforcement learning frameworks, enhancing convergence performance. Utilizing a deep reinforcement learning framework, the algorithm achieves joint decision-making optimization for computational resources, task offloading, and compression ratios, thereby minimizing the total task processing latency while satisfying transmit power and computational resource constraints. Simulation results demonstrate that the proposed scheme exhibits significant advantages over benchmark algorithms in terms of convergence speed and task processing latency.

## 1. Introduction

With the rapid development of the Internet of Things (IoT) and mobile communication technologies, the network edge is facing the dual challenges of massive terminal access and explosive data growth [1]. Traditional mobile cloud computing supports resource-constrained terminals via cloud servers, but faces a critical bottleneck: its centralized model requires long-distance data transmission to remote clouds, make it fail to meet real-time demands of applications, such as autonomous driving and remote surgery [2]. To address this issue, European Telecommunications Standards Institute (ETSI) proposed Mobile Edge Computing (MEC) architecture [3]. By sinking cloud computing capabilities to network edge nodes, MEC enables nearby services for terminal devices, effectively solving the latency bottleneck of traditional architectures. Recently, MEC architecture demonstrates significant application value in fields such as smart healthcare, intelligent driving, and environmental monitoring [4].

However, with the surging number of IoT devices and the scarcity of spectrum resources, conventional orthogonal multiple access (OMA) techniques are increasingly unable to satisfy the dual requirements of low latency and low energy consumption in MEC networks. Fortunately, non-orthogonal multiple access (NOMA) has been regarded as an emerging technology. By enabling multiple users to share the same time-frequency resources, NOMA can significantly enhance the system spectral efficiency [5]. Accordingly, the synergy between NOMA and MEC (referred to as NOMA-MEC) offers significant advantages, such as enabling massive connectivity, achieving low latency, enhancing energy efficiency, and providing the flexibility [6,7,8].

While the NOMA-MEC technology enhances edge device processing capabilities, rapid growth in sensors and service demands creates critical resource scheduling challenges [9,10,11]. In especial, vision-based applications particularly strain wireless networks due to frequent transmission of high-resolution images and videos [12]. Compression-based offloading strategies address this issue by compressing data before transmission to edge servers. This reduces data size, saving energy and minimizing latency [13]. Therefore, optimal compression and resource allocation strategies are essential for balancing communication efficiency with computational performance in edge computing networks.

### 1.1. Related Works

#### 1.1.1. NOMA-Enabled MEC

Recently, numerous studies have concentrated on optimizing the latency-energy performance of MEC systems with OMA/NOMA. For example, in heterogeneous cellular MEC networks, ref. [14] considered both task accuracy requirements and the parallel computing capabilities of MEC servers, aiming to maximize the system efficiency by jointly optimizing computation offloading strategies and resource allocation. The authors of [15] designed a collaborative optimization framework for UAV trajectory, task offloading, and communication resource allocation, to minimize the computational latency and energy consumption of tasks in MEC systems. Moreover, considering the uncertainties in real-world MEC networks, ref. [16] investigated energy-efficient computation offloading strategies. For NOMA-MEC networks, the work [17] proposed a joint resource management method based on DRL frameworks to reduce task computation latency. Furthermore, ref. [18] achieved the minimization of computational energy consumption in NOMA-MEC networks, by jointly optimizing power allocation and the time slot duration. For multi-cell NOMA-MEC networks, the authors of [19] introduced a game theory-based resource optimization approach that minimizes the weighted sum of delay and energy consumption. However, these works have not exploited data compression for computation offloading.

#### 1.1.2. Compression-Assisted MEC

While a high compression rate can reduce channel resource requirement in task offloading, it frequently results in significant accuracy degradation for task services. On the other hand, the offloading with low compression rate consumes more network resources and might even lead to transmission failures caused by excessive latency. Therefore, in order to strike a balance between communication efficiency and computation performance in edge computing networks, it is vital to develop an appropriate compression offloading and resource allocation strategy. Existing research has made some progress in tackling these challenges. For instance, to minimize the task energy consumption, ref. [20] proposed a collaborative optimization scheme for data compression ratio and resource configuration in MEC networks. The work [21] investigated an efficient and secure multi-user multi-task computation offloading model for MEC. Moreover, for hierarchical MEC systems, ref. [22] designed a three-step framework algorithm for data compression and resource allocation, aiming to reduce the weighted system cost. For low earth orbit satellite networks, the work [23] jointly optimized the data compression and task scheduling strategy to maximize the system utilization. Furthermore, for industrial internet of things assisted by UAV, the authors of [24] proposed an energy efficient trajectory and scheduling optimization based on data compression. However, the above-mentioned work often treated compression ratio as a fixed value, one-sided benefit or apply it indiscriminately. They lack the mechanism to adaptively decide whether to compress, and to what degree, based on real-time system states.

#### 1.1.3. DRL for Continuous Control

For Deep Reinforcement Learning (DRL) algorithms, the work [25] proposed an optimization framework based on Deep Deterministic Policy Gradient (DDPG), which dynamically coordinates the offloading decisions and resource allocation strategies in MEC systems. Ref. [26] developed a hybrid DRL algorithm to minimize the weighted cost of energy consumption and service latency, by optimizing the offloading strategy in a wireless power transfer-aided MEC network [27]. Additionally, ref. [28] minimized the total computational latency for multi-access MEC network by integrating a DRL framework with a coordination mechanism. However, these existing DRL algorithms easily suffer from accumulated bias in value function estimation. Moreover, in traditional DRL algorithms, overestimation can also lead to oscillations in policy updates, while underestimation may reduce the convergence speed, both of which cause low learning efficiency of the algorithms in complex and dynamic environments.

### 1.2. Motivations and Contributions

To cope with the above challenges, this paper constructs a NOMA-MEC network architecture based on data compression. By establishing a joint optimization model of computational resources, offloading strategies, and compression ratios in a dynamic environment, the total task processing latency is minimized under constraints such as the user’s battery capacity and the MEC server’s computing power, by exploiting a DRL framework based on Softmax Deep Double Deterministic Policy Gradient (SD3). Specifically, a composite state space integrating multi-user task specifications, real-time battery levels, and time-varying channel gains is firstly designed, and then a joint action space capable of simultaneously outputting continuous-valued compression ratios, offloading ratios, and server computational resource allocations is constructed. Finally a reward function incorporating penalty terms for constraint violations is designed. The main contribution of this paper is given as follows.

- To improve the offloading efficiency of multi-user MEC scenarios, a NOMA-MEC network architecture based on data compression processing is proposed. This architecture comprehensively considers practical factors such as users’ battery capacity, task computation latency, and computational resource limitations. A total task processing latency minimization optimization problem of computational resources, offloading strategies, and data compression ratios is formulated.
- A DRL framework based on SD3 is proposed to tackle the joint optimization of offloading proportion, compression ratio, and computation resource. Unlike methods that update optimization variables alternately, our approach outputs all variables simultaneously, enabling coordinated decisions that minimize the total task processing latency in NOMA-MEC networks. The framework exhibits strong adaptability to dynamic network environments and user mobility, providing valuable practical insights for real-world deployment.
- We compare and discuss the effects of computing frequency of the edge server, task data size, number of users, and bandwidth on the total long-term task latency. Furthermore, extensive simulation results show that our algorithm outperforms other benchmark algorithms concerning the total long-term task latency and the convergence speed. The findings offer important implications for the design of future wireless communication systems, particularly in MEC scenarios requiring ultra-low latency and high reliability services.

The structure of the paper is outlined as follows. In Section 2, the system model and the associated optimization problem are presented. Then, in Section 4, the optimization problem is reformulated as a MDP, and more details about the design of the proposed SD3 algorithm are introduced. In Section 5, numerical simulations are given, as well as a comparative analysis of the results. Finally, Section 6 is the conclusion of this paper.

## 2. System Model

**Figure 1:**
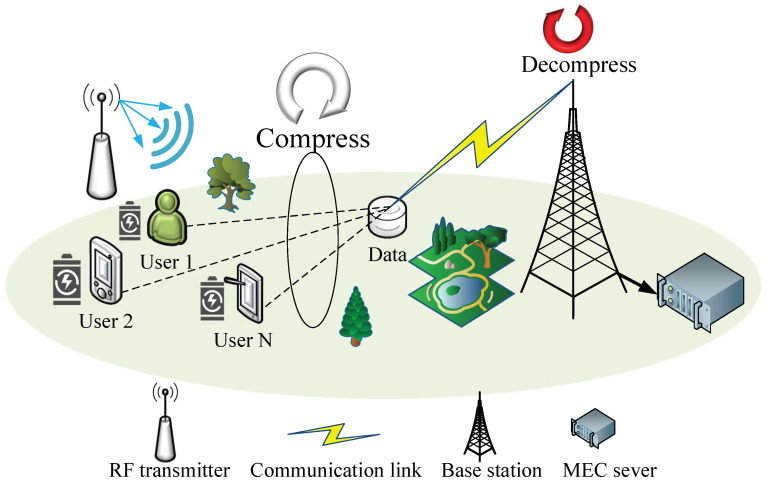
The NOMA-MEC network model.

As shown in Figure 1, we consider a NOMA-MEC network consisting of *N* users and a BS equipped with an edge server. It is assumed that all users and the base station are equipped with a single antenna. The set of users is denoted as N={1,…,n,…,N}. Each user is equipped with a rechargeable battery and can harvest Radio Frequency (RF) energy from the surrounding environment to sustain device operation. In the system model, the total time is divided into *T* equal-length time slots, each with a duration of τ, and each time slot is identified by the index t∈{1,2,…,T}. It is assumed that Channel State Information (CSI) remains quasi-static within each time slot.

### 2.1. Communication Model

At each time slot *t*, *N* users utilize NOMA to offload their computation tasks to the BS concurrently [29]. The BS receives and competes the offloaded tasks within each time slot, then returns the computation results to the served users. Assuming the channel gains between all users and the base station are sorted in descending order, i.e., $h_{1}\left(t\right)\geq h_{2}\left(t\right)\geq \cdots \geq h_{N}\left(t\right)$. Accordingly, the achievable offloading rate for user *n* is given by

(1) $$R_{n}\left(t\right)=B\log_{2}\left(1+\frac{P_{n}h_{n}\left(t\right)}{\sum_{k=n+1}^{N}P_{k}h_{k}\left(t\right)+\sigma^{2}}\right),$$

where *B* is the channel bandwidth, $P_{n}$ is the transmit power of user *n*, $\sigma^{2}$ is the noise power. When transmission rates are limited, the system may either increase the compression ratio to reduce data transmission latency or allocate additional computing resources to minimize overall computational latency.

### 2.2. Computation Model

Due to limited computational capabilities, each user adopts a partial offloading scheme, whereby a portion of its task can be offloaded to the edge server for computation, with the remaining portion executed locally. The computational task of user *n* can be represented as $I_{n}\left(t\right)=\left\{D_{n}\left(t\right),C_{n}\left(t\right)\right\}$, where $D_{n}\left(t\right)$ denotes the task size (in bits) of user *n*’s task, and $C_{n}\left(t\right)$ represents the number of CPU cycles required to compute each bit of task data (cycles per bit). Let $x_{n}\left(t\right)\in \left[0,1\right]$ be the task offloading ratio of user *n* in time slot *t*, meaning that a task amount of $x_{n}\left(t\right)D_{n}\left(t\right)$ is offloaded to the BS, and the remaining task amount of $\left(1-x_{n}\left(t\right)\right)D_{n}\left(t\right)$ is executed locally at user *n*.

#### 2.2.1. Local Computing

In the local computing mode, the execution latency of user *n* at time slot *t* is given as

(2) $$T_{n}^{L}\left(t\right)=\frac{D_{n}\left(t\right)\left(1-x_{n}\left(t\right)\right)C_{n}\left(t\right)}{f_{n}^{l}\left(t\right)},$$

where $f_{n}^{l}\left(t\right)$ is the local CPU computing frequency.

Similarly, the energy consumption by user *n* to handle the task locally is represented as

(3) $$E_{n}^{L}\left(t\right)=D_{n}\left(t\right)\left(1-x_{n}\left(t\right)\right)C_{n}\left(t\right)\kappa_{c},$$

where $\kappa_{c}$ indicates the energy consumption of the CPU per cycle for computing.

#### 2.2.2. Edge Computing

The task offloading process consists of three key phases: the task transmission phase, the task execution phase, and the result return phase. It is worth noting that when the MEC server returns the computation results to the user, the computation results are usually far smaller in magnitude than the input task data, so the delay and energy consumption of this phase can be neglected [30].

##### Task Transmission Stage

When tasks are offloaded to the MEC server, the user firstly performs local compression processing on the task data to be offloaded. Then, the compressed task data is transmitted to the base station. Upon receiving the compressed data, the base station decompresses it before performing subsequent computation and processing. In this paper, taking into account factors such as data integrity, complexity and real-time performance trade-offs, as well as compatibility, Huffman compression model is adopted [31]. Let $\delta_{comp}\in \left(0,1\right]$ denote compression ratio, meaning one bit of raw data is compressed to $\delta_{comp}$ bits; $\delta_{comp}$ is a fixed parameter determined solely by the chosen compression method. Additionally, we adopt a partial compression scheme [21], where only a fraction $b_{n}\left(t\right)\in \left(0,1\right]$ of the raw data for user *n* is compressed, while the rest is transmitted uncompressed, $b_{n}\left(t\right)=0$ and $b_{n}\left(t\right)=1$ represent no compression and complete compression, respectively. Specifically, a tractable model can be used [20], where the required CPU cycles for compressing 1-bit of raw task for user *n* can be approximated with an exponential function of the task compression ratio $\delta_{comp}$ as [22]

(4) $$C^{comp}=e^{\epsilon \delta_{comp}}-e^{\epsilon },$$

where ϵ is a positive constant depending on the data compression method. Specially, when $\delta_{comp}=1$, the required CPU cycle is zero which indicates no data compression operation is executed. Correspondingly, the delay and energy consumption caused by the compression of user *n* in time slot *t* can be expressed as

(5) $$T_{n}^{Comp}\left(t\right)=\frac{D_{n}\left(t\right)x_{n}\left(t\right)b_{n}\left(t\right)C^{comp}}{f_{n}^{l}\left(t\right)},$$

(6) $$E_{n}^{Comp}\left(t\right)=D_{n}\left(t\right)x_{n}\left(t\right)b_{n}\left(t\right)C^{comp}\kappa_{comp},$$

respectively, where $\kappa_{comp}$ is the energy consumption per cycle for data compression. It is assumed that the MEC server applies the same compression technique for decompression, so the corresponding latency for decompression is given as

(7) $$T_{n}^{Decomp}\left(t\right)=\frac{D_{n}\left(t\right)x_{n}\left(t\right)b_{n}\left(t\right)C^{comp}}{f_{n}^{e}\left(t\right)},$$

where $f_{n}^{e}\left(t\right)$ represents the CPU computing frequency assigned by the MEC server.

Moreover, at time slot *t*, the transmission latency and energy consumption for user *n* is given by

(8) $$T_{n}^{R}\left(t\right)=\frac{D_{n}\left(t\right)x_{n}\left(t\right)\left(1-b_{n}\left(t\right)\right)+D_{n}\left(t\right)x_{n}\left(t\right)b_{n}\left(t\right)\delta_{comp}}{R_{n}\left(t\right)},$$

(9) $$E_{n}^{R}\left(t\right)=P_{n}T_{n}^{R}\left(t\right),$$

respectively.

##### Task Execution Stage

After decompression, the MEC server executes the offloaded computation task. For user *n*, the latency of the MEC server executing task at time slot *t* is written as

(10) $$T_{n}^{C}\left(t\right)=\frac{D_{n}\left(t\right)x_{n}\left(t\right)C_{n}\left(t\right)}{f_{n}^{e}\left(t\right)}.$$

Therefore, in time slot *t*, the latency incurred for completing user *n*’s offloaded computing task can be expressed as

(11) $$T_{n}^{M}\left(t\right)=T_{n}^{Comp}\left(t\right)+T_{n}^{R}\left(t\right)+T_{n}^{Decomp}\left(t\right)+T_{n}^{C}\left(t\right).$$

In summary, the total delay for user *n* to complete the computing task within time slot *t* can be expressed as

(12) $$T_{n}\left(t\right)=\max\left\{T_{n}^{L}\left(t\right),T_{n}^{M}\left(t\right)\right\}.$$

Similarly, the total energy consumed by user *n* to complete the computing task in time slot *t* can be expressed as

(13) $$E_{n}\left(t\right)=E_{n}^{L}\left(t\right)+E_{n}^{Comp}\left(t\right)+E_{n}^{R}\left(t\right).$$

### 2.3. Energy Harvesting Model

The system employs a linear energy harvesting model for wireless charging of each user device. This model comprises a RF energy transmitter and the user’s rechargeable battery [32]. Due to the varying spatial distribution of users around the transmitter across different time slots, the amount of energy harvested by each user also fluctuates. The distance $m_{n}\left(t\right)$ between a specific user *n* and the RF energy transmitter is modeled as a time series following a Markov chain, characterized by its transition probability matrix [33]. To account for practical energy conversion losses during the storage process in the battery, an energy conversion efficiency factor (denoted as η) is introduced. Consequently, the energy collected by user *n* from the RF energy transmitter during a specific time slot *t* is given by

(14) $$e_{n}\left(t\right)=\eta P_{RF}{\left(m_{n}\left(t\right)\right)}^{-\varphi }h_{RF}\tau ,$$

where $P_{RF}$ is transmit power of RF transmitter, φ indicates path loss factor, and $h_{RF}$ is antenna gain of between transmitting and receiving antennas, η is energy conversion factor.

It is presumed that at the beginning of time slot *t*, the battery energy is denoted as $B_{n}\left(t\right)$. To prevent over-discharging of the battery, the entire process is consistent with the energy causality constraint and cannot exceed the maximum battery capacity $B_{\max}$. Consequently, the battery energy of user *n* must change with

(15) $$B_{n}\left(t+1\right)=\min\left\{{\left[B_{n}\left(t\right)+e_{n}\left(t\right)-E_{n}\left(t\right)\right]}^{+},B_{\max}\right\},$$

where $B_{\max}$ is the maximal battery capacity of each user, and ${\left[x\right]}^{+}=\max\left\{0,x\right\}$.

## 3. Problem Formulation

In this paper, the goal is to minimize the total long-term task processing latency through the joint optimization of offloading proportion $X\left(t\right)=\{x_{1}\left(t\right),x_{2}\left(t\right),\ldots ,x_{N}\left(t\right)\}$, compression ratio $B\left(t\right)=\{b_{1}\left(t\right),b_{2}\left(t\right),\ldots ,b_{N}\left(t\right)\}$, and computation resource allocation at the MEC server $F\left(t\right)=\{f_{1}^{e}\left(t\right),f_{2}^{e}\left(t\right),\ldots ,f_{N}^{e}\left(t\right)\}$. Accordingly, the optimization problem is formulated as

(16) $$\begin{array}{cc} \; & P1:\min_{\left\{X(t),B(t),F(t)\right\}}\frac{1}{T}\sum_{t=1}^{T}\sum_{n\in N}T_{n}\left(t\right)\\ s.t.\; & C1:\;E_{n}\left(t\right)\leq B_{n}\left(t\right)+e_{n}\left(t\right),n\in N,\forall t,\\ \; & C2:\;T_{n}\left(t\right)\leq \tau ,n\in N,\forall t,\\ \; & C3:\;\sum_{n\in N}f_{n}^{e}\left(t\right)\leq F_{\max},\forall t,\\ \; & C4:\;0\leq x_{n}\left(t\right)\leq 1,n\in N,\forall t,\\ \; & C5:\;0\leq b_{n}\left(t\right)\leq 1,n\in N,\forall t, \end{array}$$

where $F_{\max}$ represents the maximal computing frequency of the MEC server, C1 indicates the energy causality constraint for each user. To avoid the task queue congestion, C2 restricts that the task processing is accomplished within each time slot. In addition, C3–C5 are the constraints for computing resources, offloading proportion, and compression ratio, respectively.

It is evident that Problem P1 is a non-convex optimization problem. The non-convexity stems from the complex coupling relationships among the optimization variables and the presence of nonlinear terms in the objective function. Traditional convex optimization methods cannot handle such non-convex structures and thus fail. While heuristic algorithms can obtain feasible solutions, they are easily trapped in local optima. Decomposition–coordination methods, due to the strong coupling between variables, result in cyclic dependencies among subproblems, making it difficult for traditional algorithms to solve it directly. Furthermore, to solve the formulated optimization problem, considering the dynamic network environment, this paper proposes a DRL framework based on the SD3 algorithm. This framework directly addresses the non-convex problem through adaptive learning to obtain optimal resource allocation decisions. The process of solving the optimization problem with this algorithm will be introduced next.

## 4. Deep Reinforcement Learning Framework Based SD3 Algorithm

To address these challenges, this paper employs a DRL framework to find the optimal resource allocation decision strategies. Specifically, first, the problem P1 is modeled as a MDP, with relevant definitions provided for the model. Subsequently, a SD3 algorithm is proposed to solve the optimization problem.

### 4.1. Markov Decision Process Modeling

In the optimization problem P1, the decisions regarding the offloading proportion of computing tasks, compression ratio, and computational resource allocation, are only relevant to the current state, but not to the past state, which is a typical MDP problem, consisting of three main elements as follows.

#### 4.1.1. State Space

At the beginning of time slot *t*, the neural network receives as input the current system state, including the task and energy status of all users and the considered network status. Specifically,

(17) $$s_{t}=\left\{L(t),E(t),H(t)\right\},$$

where $L\left(t\right)={\left\{D_{n}\left(t\right),C_{n}\left(t\right)\right\}}_{n\in N}$ represents the task parameter of all users; $E\left(t\right)={\left\{B_{n}\left(t\right)\right\}}_{n\in N}$ is the remaining battery energy at each user; $H\left(t\right)={\left\{h_{n}\left(t\right)\right\}}_{n\in N}$ is the channel gains between all user and BS.

#### 4.1.2. Action Space

At time slot *t*, the action space consists of the offloading proportion, compression ratio, and computation resource allocation of the MEC server. Specifically, the action space is define as

(18) $$a_{t}=\left\{X\left(t\right),B\left(t\right),F\left(t\right)\right\}$$

where $X\left(t\right)={\left\{x_{n}\left(t\right)\right\}}_{n\in N}$ is the task offloading strategy; $B\left(t\right)={\left\{b_{n}\left(t\right)\right\}}_{n\in N}$ is the set of all users’ task data compression ratio; $F\left(t\right)={\left\{f_{n}^{e}\left(t\right)\right\}}_{n\in N}$ represents the computation resource allocation strategy at BS.

#### 4.1.3. Reward Function

The design of reward function should align with the system’s optimization objective, ensuring that the system evolves toward the minimization of total long-time task latency. Specifically,

(19) $$r_{t}=\sum_{n\in N}\left(-T_{n}\left(t\right)+r_{n}^{penalty}\left(t\right)\right),$$

where $r_{n}^{penalty}\left(t\right)$ is the penalty actions that violate constraints are penalized, reflected in the reward value, helping the agent to make better decisions, it is defined as

(20) $$r_{n}^{penalty}\left(t\right)=\left\{\begin{array}{cc} -2, & E_{n}\left(t\right)>B_{n}\left(t\right)+e_{n}\left(t\right)\;\mathrm{and}\;T_{n}\left(t\right)>\tau ;\\ -1, & E_{n}\left(t\right)>B_{n}\left(t\right)+e_{n}\left(t\right)\;\mathrm{or}\;T_{n}\left(t\right)>\tau ;\\ 0, & \mathrm{other}. \end{array}\right.$$

### 4.2. The Framework of SD3 Algorithm

**Figure 2:**
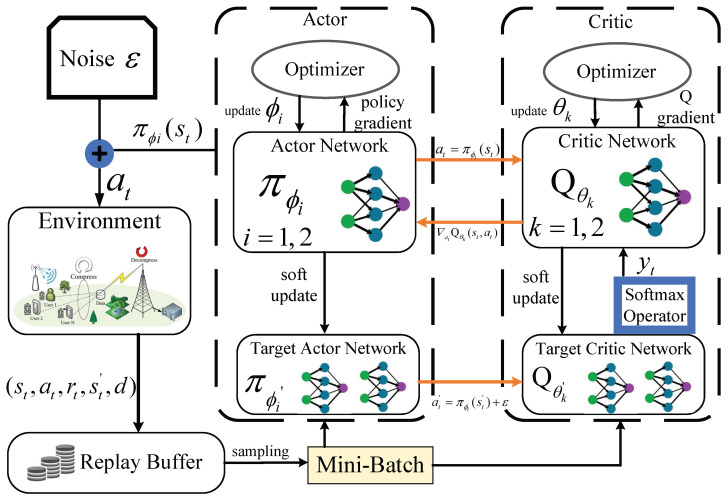
The framework of SD3 algorithm.

DRL algorithms for continuous control, notably DDPG and Twin Delayed DDPG (TD3), exhibit inherent limitations in value estimation accuracy [34]. For example, DDPG suffers from systematic overestimation bias due to maximization operations in its Q-function updates, leading to inflated action-value approximations and suboptimal policies. Although TD3 mitigates this overestimation through clipped double Q-learning and delayed policy updates, it inadvertently induces persistent underestimation bias. This secondary bias constrains exploration efficacy and ultimately degrades asymptotic performance. In contrast, the SD3 algorithm employs a dual-mechanism framework to counteract the inherent biases in value estimation. Its key contributions include (i) a Boltzmann softmax operator applied to double Q-estimators, which replaces conventional max/min operators and alleviates overestimation bias; (ii) bias-corrected targets that dynamically balance the variances between the two estimators, thus harmonizing opposing estimation errors. This design harmonizes opposing biases while preserving approximation accuracy during temporal-difference updates. Compared to TD3 and DDPG, SD3 achieves superior sample efficiency and asymptotic performance by simultaneously constraining both overestimation and underestimation biases. The synergistic integration of softmax-based regularization with variance-aware double estimation establishes a new state-of-the-art in bias-resilient continuous control [35]. In Figure 2, the framework of SD3 algorithm is given to clarify the SD3 training and execution process.

SD3 algorithm is founded upon the actor-critic framework with double actor networks $\pi_{\phi_{i}}\left(i=1,2\right)$ and double critic networks $Q_{\theta_{k}}\left(k=1,2\right)$, corresponding network function parameters are $\phi_{i}$ and $\theta_{k}$. When the agent starts learning, SD3 algorithm randomly samples $\left(s_{t},a_{t},r_{t},s_{t}^{\prime },d\right)$ from the replay buffer to form a mini-batch of *N* training data. The target actor network predicts the next actions $a_{t}^{\prime }$, and the *Q* value can be given as

(21) $$\hat{Q}\left(s_{t}^{\prime },a_{t}^{\prime }\right)=\min_{k=1,2}Q_{\theta_{k}^{\prime }}\left(s_{t}^{\prime },a_{t}^{\prime }\right).$$

In the subsequent phase, to smooth the *Q* value estimate and reduce the estimation error, this algorithm introduces the softmax operator which is defined as

(22) $${\mathrm{softmax}}_{\beta }\left(\hat{Q}\left(s_{t}^{\prime },a_{t}^{\prime }\right)\right)=\frac{E_{a_{t}^{\prime }∼p}\left[\frac{\exp\left(\beta \hat{Q}\left(s_{t}^{\prime },a_{t}^{\prime }\right)\right)\hat{Q}\left(s_{t}^{\prime },a_{t}^{\prime }\right)}{p(a_{t}^{\prime })}\right]}{E_{a_{t}^{\prime }∼p}\left[\frac{\exp(\beta \hat{Q}\left(s_{t}^{\prime },a_{t}^{\prime }\right))}{p(a_{t}^{\prime })}\right]},$$

where β is the softmax operator parameter, and $p(a_{t}^{\prime })$ represents the probability density function of a Gaussian distribution. The softmax operator serves to mitigate the impact of actions with estimation error on the results by taking a weighted average of *Q* values, thereby enhancing the stability of the policy. The target value $y_{t}$ can be calculated in conjunction with the discount factor γ as

(23) $$y_{t}=r_{t}+\gamma \left(1-d\right){\mathrm{softmax}}_{\beta }\left(\hat{Q}\left(s_{t}^{\prime },a_{t}^{\prime }\right)\right).$$

governing the agent’s policy trade-offs between immediate and future latency penalties.

The target value is employed to adjust the parameters of the critic network, allowing it to better approximate the value of the state–action pair. The loss function is designed to measure the difference between the target value and the current *Q* value, which can be represented as

(24) $$L\left(\theta_{k}\right)=\min_{\theta_{k}}\frac{1}{N}\sum_{n=1}^{N}{\left(y_{t}-Q_{\theta_{k}}\left(s_{t},a_{t}\right)\right)}^{2}.$$

The parameters of the critic network denoted as $\theta_{k}$, are iteratively adjusted using gradient descent to reduce the loss function.

Next, the goal of updating the actor network is to determine the optimal policy parameters that maximize the objective function $J(\phi_{i})$. These parameters maximize the expected return, which can be given by

(25) $$J\left(\phi_{i}\right)=\frac{1}{N}\sum_{n=1}^{N}Q_{\theta_{k}}\left(s_{t},\pi_{\phi_{i}}\left(s_{t}\right)\right).$$

Subsequently, the following expression can be substituted in order to calculate the policy gradient as

(26) $$\nabla_{\phi_{i}}J\left(\phi_{i}\right)=\frac{1}{N}\sum_{n=1}^{N}\left(\nabla_{\phi_{i}}\left(\pi \left(s_{t},\phi_{i}\right)\right)\nabla_{a_{t}}Q_{\theta_{k}}\left(s_{t},a_{t}\right){|}_{a_{t}=\pi \left(s_{t},\phi_{i}\right)}\right).$$

Finally, the target network is subject to a soft update, which allows for the implementation of a latency update strategy. This method can improve the convergence stability by minimizing the fluctuations seen in the target network. The specific procedures of the SD3 algorithm are presented in Algorithm 1.

**Algorithm 1:** SD3 algorithm for joint data compression and resource allocation
  1:Input: Environment states (channel, task, energy), neural networks  2:Output: Optimal policy for compression, offloading, resource allocation  3:Initialize SD3 agent (Actor & Critic networks), replay buffer  4:**for** each episode **do**  5:    Observe initial state $s_{t}=\left\{x_{n}\left(t\right),b_{n}\left(t\right),f_{n}^{e}\left(t\right)\right\}$  6:    **for** each time slot *t* **do**  7:        Agent selects action $a_{t}=\left\{X\left(t\right),B\left(t\right),F\left(t\right)\right\}$ using policy π  8:        Execute action:  9:        Users: Compress data $b_{n}\left(t\right)$ [Equation (4)], offload $x_{n}\left(t\right)$10:        MEC: Decompress & compute [Equations (6) and (9)]11:        Update battery with harvested RF energy [Energy Harvesting Model]12:        Calculate reward $r_{t}=\sum_{n\in N}\left(-T_{n}\left(t\right)+r_{n}^{penalty}\left(t\right)\right)$ [Equation (11)]13:        Store transition $\left(s_{t},a_{t},r_{t},s_{t+1}\right)$ in replay buffer14:        Sample mini-batch; update Critic via Softmax-smoothed Q-values: $(Q_{\mathrm{softmax}}=\tau \log\sum \exp\left(Q_{i}/\tau \right)$15:        Update Actor using deterministic policy gradient16:    **end for**17:
**end for**



### 4.3. Computational Complexity Analysis

In DRL frameworks, parameter updates are dominated by matrix multiplication operations. Considering that the dimensions of state space and action space are 3N and 4N, respectively. The SD3 algorithm adopts an *L*-layer fully connected network where $K_{l}$ denotes the number of neurons in the *l*-th layer. Compared to TD3 and DDPG, which share the same network architecture, all three algorithms have per-iteration complexity scaling as $O(B_{s}\cdot N\cdot \sum_{l=1}^{L}K_{l-1}\cdot K_{l})$ for batch size $B_{s}$. While SD3’s novel components (softmax operator and variance-corrected targets) introduce additional steps, they contribute only O(1) overhead. The per-iteration computational complexity of all algorithms grows linearly with the number of users *N*, as the dimensions of both the state and action spaces are proportional to *N*. Thus, SD3 maintains the same asymptotic complexity as its counterparts: $O(T\cdot B_{s}\cdot N\cdot \sum_{l=1}^{L}K_{l-1}\cdot K_{l})$ over *T* time frames. Empirically, this translates to a minimal runtime increase of only 5–7% per training step—a negligible cost that enables SD3’s superior bias correction and sample efficiency.

In contrast, Actor-Critic (AC) methods typically maintain separate policy and value networks with $L_{a}$ and $L_{c}$ layers respectively, resulting in higher computational complexity of $O(B_{s}\cdot N\cdot \left(\sum_{l=1}^{L_{a}}K_{l-1}^{a}K_{l}^{a}+\sum_{l=1}^{L_{c}}K_{l-1}^{c}K_{l}^{c}\right))$ due to dual-network updates. Dueling Double Deep Q-Network (D3QN) introduces dueling architecture where the final hidden layer splits into parallel advantage and value streams, adding moderate overhead with complexity $O(B_{s}\cdot N\cdot \left(\sum_{l=1}^{L-1}K_{l-1}K_{l}+2K_{L-1}K_{L}\right))$. To sum up, Table 1 shown compares the computational complexity of the proposed algorithms and traditional ones. It is clear from Table 1 that, while both AC and D3QN incur higher computational costs than SD3, they remain efficient within their respective learning paradigms. Overall, SD3 achieves advanced bias-variance trade-off without significant complexity increase, maintaining competitive efficiency among state-of-the-art DRL algorithms.

## 5. Simulation Results

The system model postulates a BS accommodating *N* = 10 users uniformly dispersed within a circular coverage area with a radius of *d*. The number of CPU cycles required per bit for computing ranges between [2000,3000] cycles/bit, and the number of CPU cycles required per bit of data for compression and decompression is 200 cycles/bit [36]. In the SD3 algorithm, the actor and critic networks consist of two hidden layers with sizes [256,128], and the activation function applied to each hidden layer is the rectified linear unit. The learning rates for the actor and critic networks are adjusted to $1\times 10^{-4}$ and $2\times 10^{-4}$, respectively. Unless otherwise specified, the basic parameters are defined in Table 2. Moreover, to assess the performance of the proposed scheme, it is compared with the following traditional baseline DRL algorithms.

- Actor–Critic (AC): The Actor–Critic algorithm combines value-based methods and policy-based methods [37]. The target *Q*-values are calculated by the same network and each time the maximum value among the current estimates of all action values from the Bellman equation is obtained. The neural network tends to accumulate positive errors, thereby causing the overestimation problem.
- Dueling Double Deep Q Network (D3QN): The value-based discrete action Deep Q Network improvement algorithm, the network for selecting actions, and the network for evaluating actions are different, and the lower value of the two is chosen [38]. This approach introduces the drawback of an overall underestimation problem.
- Deep Deterministic Policy Gradient (DDPG): The algorithm integrates deep learning with deterministic policy gradient to address issues in continuous action space problems [39]. Built upon the Actor–Critic framework, the DDPG algorithm can achieve effective offline policy learning in complex high-dimensional environments, but it is unable to avoid estimation errors.

**Figure 3:**
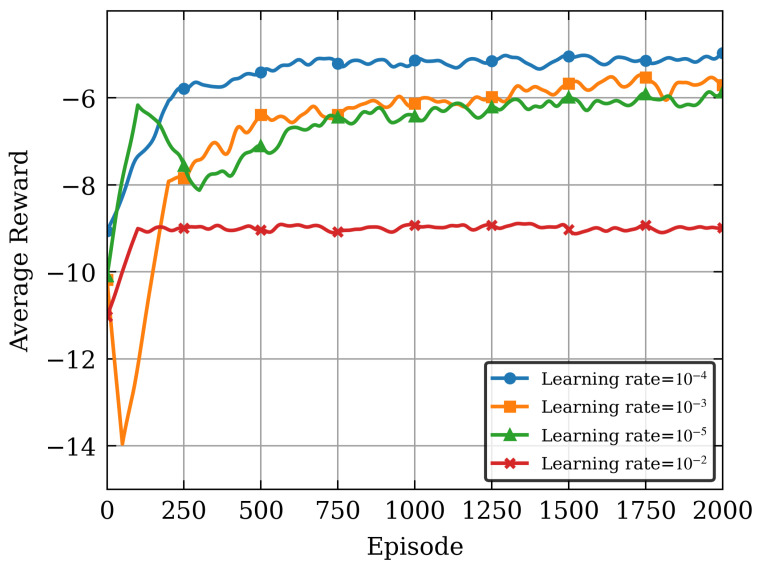
The convergence behavior under different learning rates.

Figure 3 shows the convergence behavior under different learning rates. As illustrated in Figure 3, both excessively large and small learning rates can degrade the algorithm’s convergence performance. A too-small learning rate may lead to local and low convergence, while a too-large learning rate can cause fluctuations and prevent convergence. A learning rate of $1\times 10^{-4}$ achieves approximately the best average reward. This also demonstrates that the algorithm is more sensitive to changes in learning rates. Moreover, it can also be seen that, there is a clear trade-off between the convergence speed and stability: higher learning rates offer faster convergence speed but with lower reward fluctuations, while lower learning rates provide slower learning processes but with higher reward.

**Figure 4:**
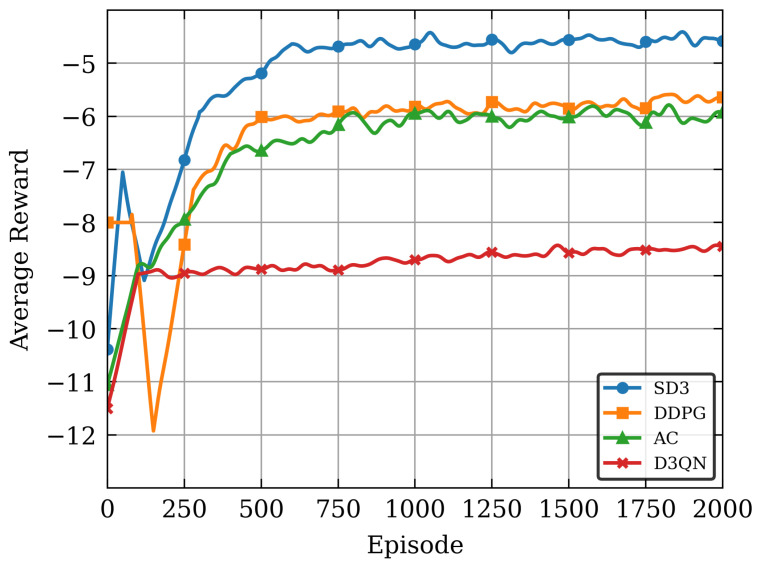
The convergence behavior of different algorithms.

Figure 4 illustrates the convergence performance of various algorithms over 2000 training episodes. The results indicate that all algorithms converge with increased training episodes. In a holistic view, SD3 algorithm outperforms other algorithms significantly, achieving higher average rewards during training. This is primarily attributable to the enhanced framework design of the SD3 algorithm and the incorporation of the softmax operation to enhance the precision of the value function. This enables SD3 algorithm to learn and optimize strategies with greater efficiency in complex environments, and avoids the impact of overestimation and underestimation biases, thereby exhibiting superior performance during training. After 2000 episodes, the AC algorithm lags behind both SD3 and DDPG algorithms in reward value, reflecting its limited capability in handling complex tasks and the online strategy framework is underutilized for five-tuple samples, the D3QN algorithm exhibits the poorest performance due to the lower accuracy of discrete actions compared to continuous actions.

**Figure 5:**
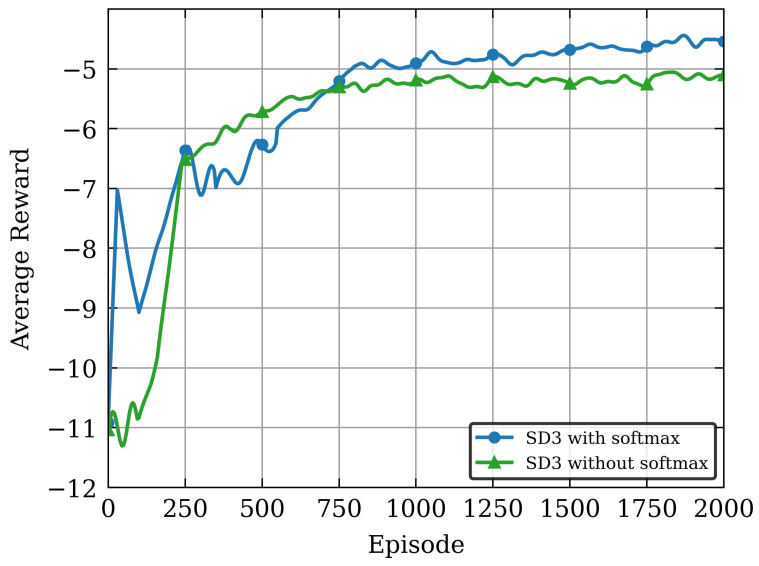
The Convergence Behaviors of SD3 Algorithm with and without the Softmax Operator.

Moreover, to validate the effectiveness of the proposed softmax operator in balancing value estimation biases of the Critic network, we conducted an ablation study on the SD3 algorithm: the convergence performance was compared under two configurations with softmax operator (SD3 with softmax) and without softmax operator (SD3 without softmax) in Figure 5. It can be seen from Figure 5 that the SD3 algorithm incorporating the softmax operator achieves higher average reward values. This result verifies the critical role of the softmax operator in suppressing value function estimation biases and enhancing policy optimization performance. This is mainly because the SD3 algorithm without the softmax operator resembles the basic dual-Critic architecture, which is more prone to accumulation of value estimation biases in dynamic and complex NOMA-MEC resource allocation environments, leading to larger training fluctuations and lower policy performance after convergence. In contrast, the softmax operator effectively balances biases between dual Q-estimators through a weighted smoothing mechanism based on the Boltzmann distribution, avoiding common overestimation or underestimation issues in traditional DDPG/TD3 algorithms, thereby improving the algorithm’s learning stability and final performance.

**Figure 6:**
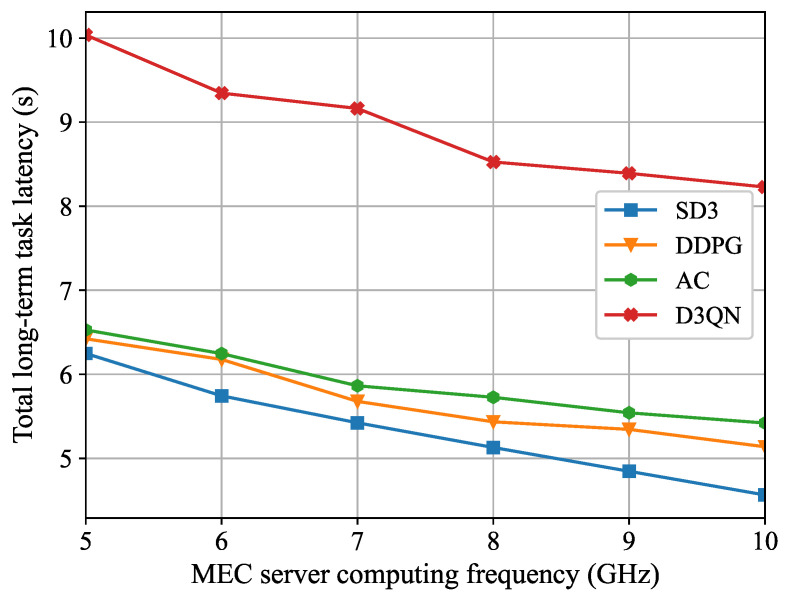
The total long-term task latency for different MEC server computing frequency.

Figure 6 demonstrates the relationship between the total long-term task latency and the MEC server computing frequency. As shown in Figure 6, the total long-term task latency diminishes as the maximum computing frequency rises. This occurs because, as the computing resources of the MEC server increase, task processing speed becomes faster and task execution latency becomes smaller. Consequently, users are more inclined to reduce task execution latency through computing offloading. In contrast to the benchmark algorithms, the total long-term task latency of the SD3 algorithm is reduced by 26.9% when the computing capability is increased from 5 GHz to 10 GHz, which still maintains the lowest average latency. As the MEC server computing capability increases, the latency decreases most significantly.

**Figure 7:**
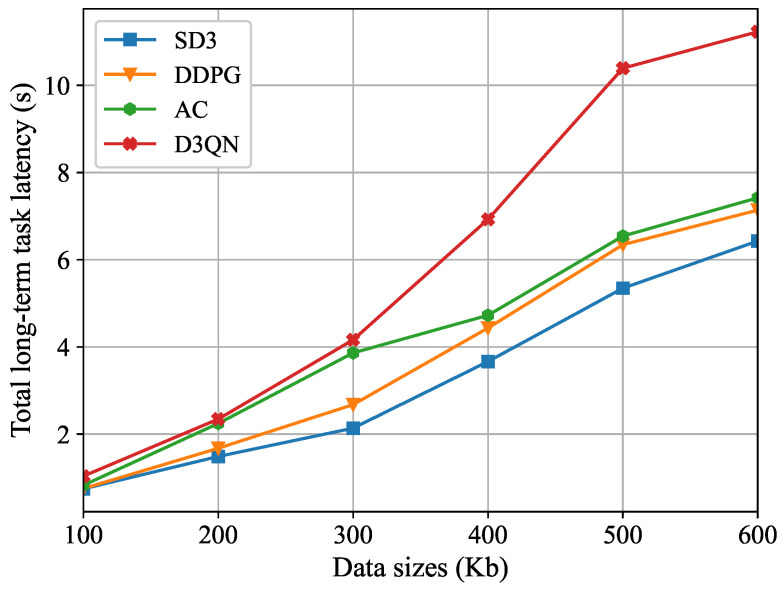
The total long-term task latency for different task data size.

Figure 7 illustrates the trend of the total long-term task latency under different task data sizes. The curve relationship indicates that as the data size of user computing tasks expands, the overall latency in the system exhibits a synchronous growth trend. This phenomenon can be attributed to two key mechanisms: First, the local computing latency increases linearly with growing data size. Second, in MEC scenarios, the transmission latency during the data transfer phase rises significantly due to enlarged packet sizes. Simulation comparison results demonstrate that, under different task data sizes, the proposed SD3 algorithm in this paper shows a marked advantage in latency control compared to other DRL-based optimization schemes.

**Figure 8:**
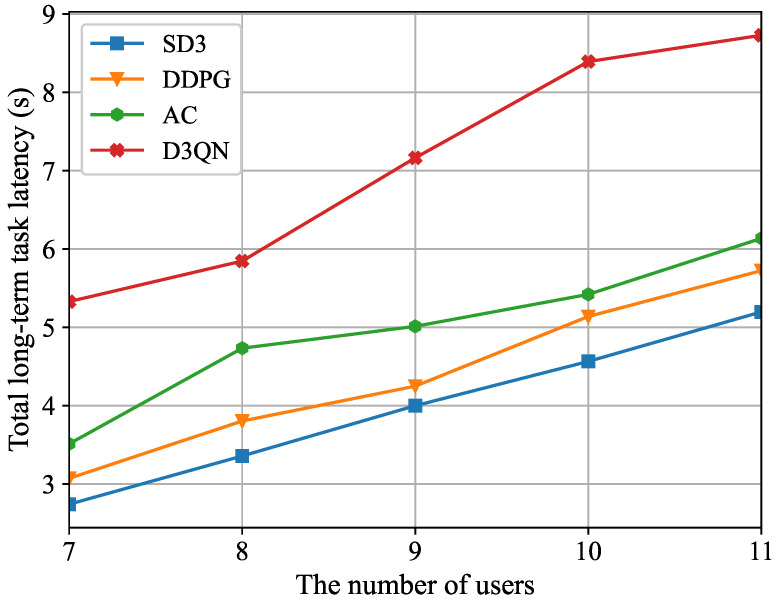
The total long-term task latency for different number of users.

Figure 8 illustrates the connection between the total long-term task latency and the number of users. It is evident that the total long-term task latency increases progressively as the number of users grows. This is due to the increase in the number of users, which results in a greater variety of processing tasks and data sizes. However, the limited computing resources of the MEC server lead to higher computing latency, and the uplink congestion further reduces the transmission rate. In addition, as the user count grows, both the state and action dimensions that the agent must handle will expand. Moreover, we also can see from Figure 8 that the SD3 algorithm is demonstrably more effective than the DDPG, AC, and D3QN algorithms in terms of the total long-term task latency, which demonstrates the robustness of the SD3 algorithm in dynamic environments.

**Figure 9:**
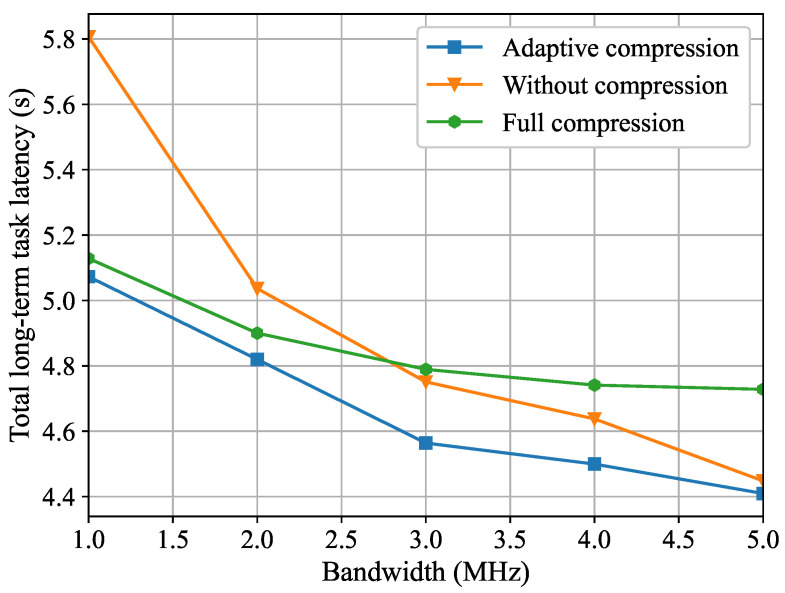
The total long-term task latency for different bandwidths.

In Figure 9, two benchmark schemes are introduced: Full compression and without compression. From Figure 9, we can observe that, when the available bandwidth is limited, the full compression scheme will perform better than the scheme without compression. However, when the bandwidth reaches a certain value, the performance of the scheme without compression will be better than that of the full compression scheme. This is because when the bandwidth resources are sufficient, the transmission rate is already high enough, and the compression processing will add an additional burden to the transmission latency. Moreover, we also can see that the proposed adaptive compression scheme can dynamically modify the compression ratio in the offloading task according to the different channel bandwidths, thereby exhibiting superior performance compared to the two benchmark schemes.

**Figure 10:**
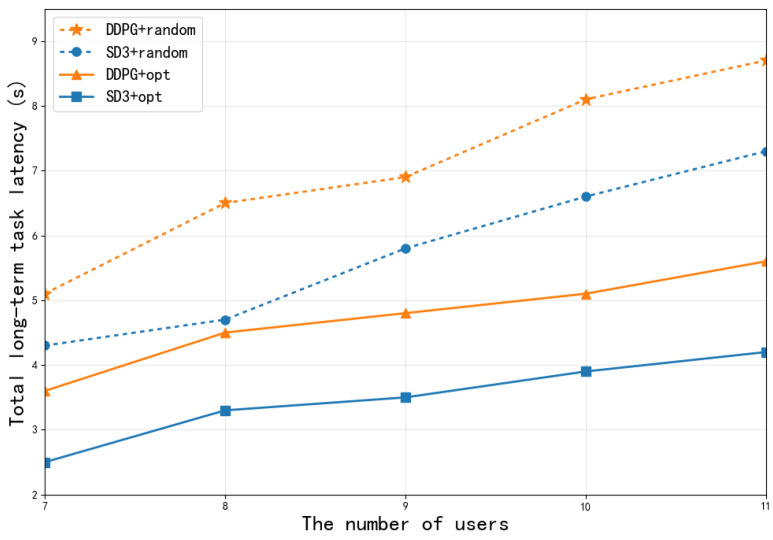
The total long-term task latency for different schemes.

To further dissect the source of performance improvement of the SD3 algorithm, Figure 10 compares the performance of the complete SD3 algorithm (SD3 + opt, i.e., equipped with both the softmax operator and cooperative optimization mechanism) and baseline algorithms with random optimization strategies (DDPG + random, SD3 + random, DDPG + opt) under different numbers of users. As shown, with increasing user numbers, the system delays of all algorithms show an upward trend due to intensified competition for computing and communication resources. However, the complete SD3 algorithm (SD3 + opt) achieves the lowest task processing delay across all user scales, significantly outperforming other configurations. Notably, compared to SD3 with only optimization (SD3 + random), SD3 with the complete optimization mechanism (SD3 + opt) shows significant performance improvement. This clearly indicates that the joint effect of the softmax operator and the compression-offloading-resource cooperative optimization mechanism is the core of the algorithm’s superiority. The softmax operator ensures stable and efficient learning, while the cooperative optimization mechanism enables adaptive optimal decision-making in dynamic multi-user environments. Their combined action significantly reduces the system’s long-term task processing delay.

## 6. Conclusions

This study tackles the problem of minimizing the total time-long latency for computational tasks within NOMA-MEC networks. Data compression techniques are introduced during task offloading to MEC servers to significantly reduce communication latency. A joint optimization framework is established, integrating computational resource allocation, offloading decisions, and data compression ratio selection. To efficiently solve this complex problem, we propose the SD3 algorithm with Softmax exploration. This novel approach substantially enhances convergence performance by effectively mitigating the overestimation and underestimation biases common in conventional reinforcement learning frameworks. Comprehensive experimental results demonstrate that the proposed SD3 algorithm outperforms benchmark methods, achieving significant improvements in convergence speed and reducing total system latency. This work provides a robust solution for enhancing task processing efficiency in next-generation MEC systems.

While the SD3-based joint optimization framework delivers remarkable latency reduction in NOMA-MEC networks, it presents notable limitations. Algorithmic hurdles include the dual-actor dual-critic architecture, which elevates training complexity by requiring four parallel network updates per iteration, sensitivity to reward function design, and scalability bottlenecks arising from linearly expanding state/action spaces. Practically, deployment necessitates GPU/NPU-equipped edge servers, lightweight terminal codecs, NOMA modules, and millisecond-level CSI feedback. In future, we will focus on three key issues: developing small-scale prototypes, exploring distributed DRL for enhanced scalability, and long-term integration of semantic compression, cross-layer orchestration, and B5G/6G standards to enable operator edge-cloud deployment.

## Figures and Tables

**Table 1:** Computational Complexity Comparison of DRL Algorithms.

Algorithm	Per-Iteration Complexity
SD3	$O(B_{s}\cdot N\cdot \sum_{l=1}^{L}K_{l-1}K_{l})$
TD3	$O(B_{s}\cdot N\cdot \sum_{l=1}^{L}K_{l-1}K_{l})$
DDPG	$O(B_{s}\cdot N\cdot \sum_{l=1}^{L}K_{l-1}K_{l})$
AC	$O(B_{s}\cdot N\cdot \left(\sum_{l=1}^{L_{a}}K_{l-1}^{a}K_{l}^{a}+\sum_{l=1}^{L_{c}}K_{l-1}^{c}K_{l}^{c}\right))$
D3QN	$O(B_{s}\cdot N\cdot \left(\sum_{l=1}^{L-1}K_{l-1}K_{l}+2K_{L-1}K_{L}\right))$

**Table 2:** Simulation Parameters.

Parameters	Symbol	Value
Total time	*T*	100 s
Time frame length	τ	1 s
Coverage area radius	*d*	100 m
Bandwidth	*B*	3 MHz
Noise power spectral density	$N_{0}$	−174 dBm
User transmit power	$P_{n}$	(0, 23) dBm
MEC server computing frequency	$F_{max}$	10 GHz
User CPU frequency	$f_{n}^{l}$	[1, 1.5] GHz
Task data size	$D_{n}\left(t\right)$	[100, 600] Kb
Compression coefficient	$\delta_{n}$	0.1
Softmax parameter	β	0.003
Discount factor	γ	0.99
Path loss index	φ	2
Energy efficiency conversion factor	η	0.51

## Data Availability

The original contributions presented in this study are included in the article. Further inquiries can be directed to the corresponding authors.

## Abbreviations

IoT	Internet of Thing
MEC	Mobile Edge Computing
UAV	Unmanned Aerial Vehicle
DDPG	Deep Deterministic Policy Gradient
DRL	Deep Reinforcement Learning
EH	Energy Harvesting
NOMA	Non-Orthogonal Multiple Access
SD3	Softmax Deep Double Deterministic Policy Gradient
MDP	Markov decision process
BS	Base Station
RF	Radio Frequency
TD3	Twin Delayed Deep Deterministic Policy Gradient
D3Q	Dueling Double Deep Q Network
AC	Actor-Critic
CSI	Channel State Information

## References

[1] Yalli J.S., Hasan M.H., Badawi A.A. (2024). Internet of things (IoT): Origins, embedded technologies, smart applications, and its growth in the last decade. IEEE Access.

[2] Jiang H., Dai X., Xiao Z., Iyengar A. (2022). Joint task offloading and resource allocation for energy-constrained mobile edge computing. IEEE Trans. Mob. Comput..

[3] Chen C.-L., Brinton C.G., Aggarwal V. (2023). Latency minimization for mobile edge computing networks. IEEE Trans. Mob. Comput..

[4] Zhu X., Ma F., Ding F., Guo Z., Yang J., Yu K. (2024). A low-latency edge computation offloading scheme for trust evaluation in finance-level artificial intelligence of things. IEEE Internet Things J..

[5] Ding Z., Lei X., Karagiannidis G.K., Schober R., Yuan J., Bhargava V.K. (2017). A survey on non-orthogonal multiple access for 5G networks: Research challenges and future trends. IEEE J. Sel. Areas Commun..

[6] Kiani A., Ansari N. (2018). Edge computing aware NOMA for 5G networks. IEEE Internet Things J..

[7] Gui Y., Lu H., Wu F., Chen C.W. (2021). Robust video broadcast for users with heterogeneous resolution in mobile networks. IEEE Trans. Mob. Comput..

[8] Dong X., Li X., Yue X., Xiang W. (2020). Performance analysis of cooperative NOMA based intelligent mobile edge computing system. China Commun..

[9] Alishahi M., Fortier P., Zeng M., Pham Q.-V., Huynh-The T. (2025). Total computational bits maximization for STAR-RIS aided wireless power transfer mobile edge computing networks: TDMA or NOMA?. IEEE Trans. Veh. Technol..

[10] Park H.J., Kim H.W., Chae S.H. (2023). Deep-learning-based resource allocation for transmit power minimization in uplink NOMA IoT cellular networks. IEEE Trans. Cogn. Commun. Netw..

[11] Hao Y., Hu L., Chen M. (2023). Joint sensing adaptation and model placement in 6G fabric computing. IEEE J. Sel. Areas Commun..

[12] Ren J., Ruan Y., Yu G. (2019). Data transmission in mobile edge networks: Whether and where to compress?. IEEE Commun. Lett..

[13] Wang J., Zhang J., Ding C., Zhang H., Lin M., Wang J. (2020). Joint optimization of transmission bandwidth allocation and data compression for mobile-edge computing systems. IEEE Commun. Lett..

[14] Zheng Y., Zhang T., Loo J., Wang Y., Nallanathan A. (2023). Joint computing offloading and resource allocation for classification intelligent tasks in MEC systems. arXiv.

[15] Zhao N., Ye Z., Pei Y., Liang Y.-C., Niyato D. (2022). Multi-agent deep reinforcement learning for task offloading in UAV-assisted mobile edge computing. IEEE Trans. Wirel. Commun..

[16] Ji T., Luo C., Yu L., Wang Q., Chen S., Thapa A., Li P. (2022). Energy-efficient computation offloading in mobile edge computing systems with uncertainties. IEEE Trans. Wirel. Commun..

[17] Wang J., Wang Y., Cheng P., Yu K., Xiang W. (2023). DDPG-based joint resource management for latency minimization in NOMA-MEC networks. IEEE Commun. Lett..

[18] Ding Z., Xu D., Schober R., Poor H.V. (2022). Hybrid NOMA offloading in multi-user MEC networks. IEEE Trans. Wireless Commun..

[19] Wu L., Sun P., Chen H., Zuo Y., Zhou Y., Yang Y. (2024). NOMA-enabled multiuser offloading in multicell edge computing networks: A coalition game based approach. IEEE Trans. Netw. Sci. Eng..

[20] Cheng K., Fang X., Wang X. (2023). Energy efficient edge computing and data compression collaboration scheme for UAV-assisted network. IEEE Trans. Veh. Technol..

[21] Elgendy I.A., Zhang W.-Z., Zeng Y., He H., Tian Y.-C., Yang Y. (2020). Efficient and secure multi-user multi-task computation offloading for mobile-edge computing in mobile IoT networks. IEEE Trans. Netw. Serv. Manag..

[22] Nguyen T.T., Ha V.N., Le L.B., Schober R. (2020). Joint data compression and computation offloading in hierarchical fog-cloud systems. IEEE Trans. Wirel. Commun..

[23] He L., Li S., Jia Z., Wang J., Han Z. (2025). Joint data compression and task scheduling for LEO satellite networks. IEEE Trans. Veh. Technol..

[24] Li B., Zhu X., Wang J. (2025). Robust trajectory design and task scheduling with data compression in industrial internet of things assisted by UAV. IEEE Internet Things J..

[25] Engidayehu S.B., Mahboob T., Chung M.Y. Deep reinforcement learning-based task offloading and resource allocation in MEC-enabled wireless networks. Proceedings of the 2022 27th Asia Pacific Conference on Communications (APCC).

[26] Hu H., Wu D., Zhou F., Zhu X., Hu R.Q., Zhu H. (2023). Intelligent resource allocation for edge-cloud collaborative networks: A hybrid DDPG-D3QN approach. IEEE Trans. Veh. Technol..

[27] Hu H., Wang Q., Hu R.Q., Zhu H. (2021). Mobility-aware offloading and resource allocation in a MEC-enabled IoT network with energy harvesting. IEEE Internet Things J..

[28] Zheng K., Jiang G., Liu X., Chi K., Yao X., Liu J. (2023). DRL-based offloading for computation delay minimization in wireless-powered multi-access edge computing. IEEE Trans. Commun..

[29] Irum T., Ejaz M.U., Elkashlan M. Minimizing task offloading delay in NOMA-MEC wireless systems. Proceedings of the 2022 4th Global Power, Energy and Communication Conference (GPECOM).

[30] Sheng M., Wang Y., Wang X., Li J. (2020). Energy-efficient multiuser partial computation offloading with collaboration of terminals, radio access network, and edge server. IEEE Trans. Commun..

[31] Nasif A.S., Othman Z.A., Sani N.S., Hasan M.K., Abudaqqa Y. (2024). Huffman Deep Compression of Edge Node Data for Reducing IoT Network Traffic. IEEE Access.

[32] Li Y., Wang F., Zhang X., Guo S. (2023). IRS-based MEC for delay-constrained QoS over RF-powered 6G mobile wireless networks. IEEE Trans. Veh. Technol..

[33] Yuan F., Zhang Q.T., Jin S., Zhu H. (2015). Optimal harvest-use-store strategy for energy harvesting wireless systems. IEEE Trans. Wirel. Commun..

[34] Arulkumaran K., Deisenroth M.P., Brundage M., Bharath A.A. (2017). Deep reinforcement learning: A brief survey. IEEE Signal Process. Mag..

[35] Pan L., Cai Q., Huang L. (2020). Softmax deep double deterministic policy gradients. Adv. Neural Inf. Process. Syst..

[36] Cao X., Gan L., Pan H., Dang X. (2023). Computation bits maximization in data compression-assisted MEC networks. Phys. Commun..

[37] Geng L., Zhao H., Wang J., Kaushik A., Yuan S., Feng W. (2023). Deep-reinforcement-learning-based distributed computation offloading in vehicular edge computing networks. IEEE Internet Things J..

[38] Zhao N., Liang Y.-C., Niyato D., Pei Y., Wu M., Jiang Y. (2019). Deep reinforcement learning for user association and resource allocation in heterogeneous cellular networks. IEEE Trans. Wirel. Commun..

[39] Yang S., Liu J., Zhang F., Li F., Chen X., Fu X. (2022). Caching-enabled computation offloading in multi-region MEC network via deep reinforcement learning. IEEE Internet Things J..

